# Supplementary material for: Effects of Melatonin and 3,5,3′-Triiodothyronine on the Development of Rat Granulosa Cells
Source: Nutrients. 2024 Sep 13;16(18):3085. doi: 10.3390/nu16183085 (PMC11435325; doi:10.3390/nu16183085)
Supplement: Supplementary file 1 [file nutrients-16-03085-s001.zip › nutrients-3155750-supplementary.pdf]

Supplementary material

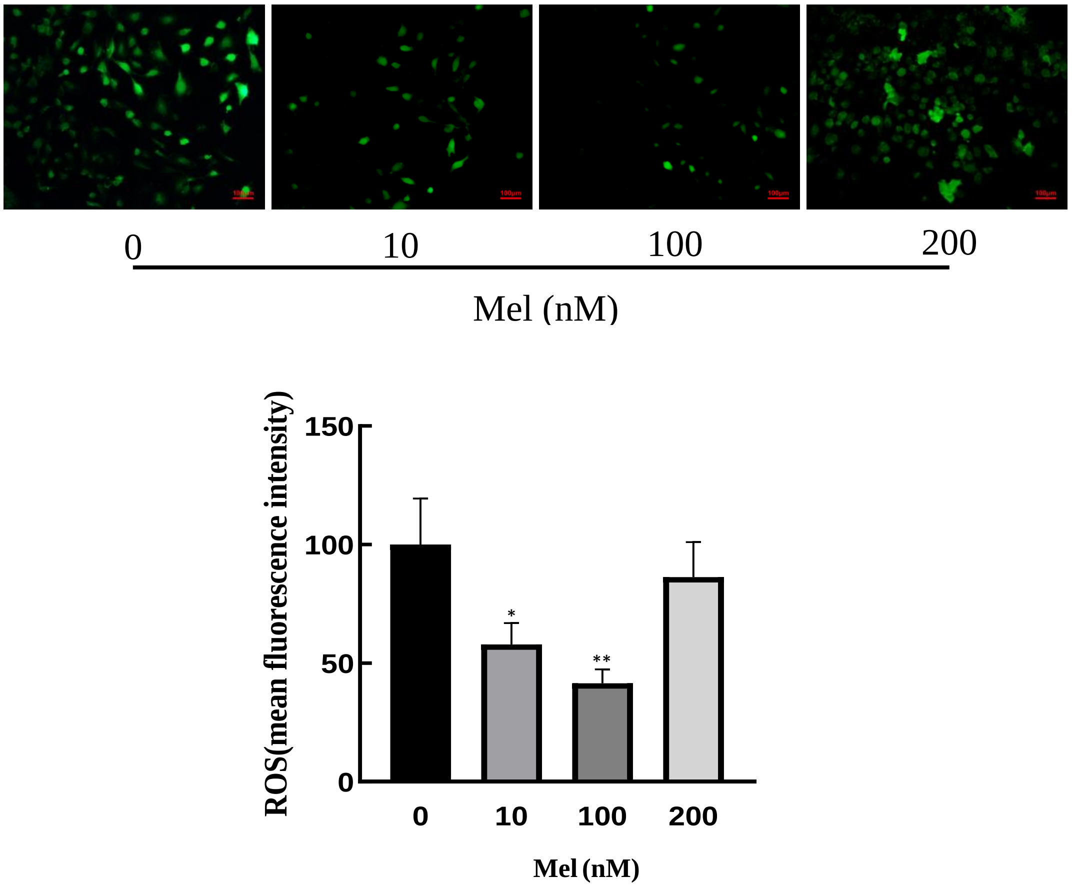

Figure S1. Effects of melatonin on ROS in rat granulosa cells. After granulosa cells were induced by H_2O_2 (100 μM) for 1 h, granulosa cells were cultured with different concentrations of melatonin (0, 10, 100 and 200 nM) for 48 h, and the ROS levels were measured. $^{**}P < 0.01$, $^{*}P < 0.05$, compared with 0 nM group. Scale bars = 100 μm.

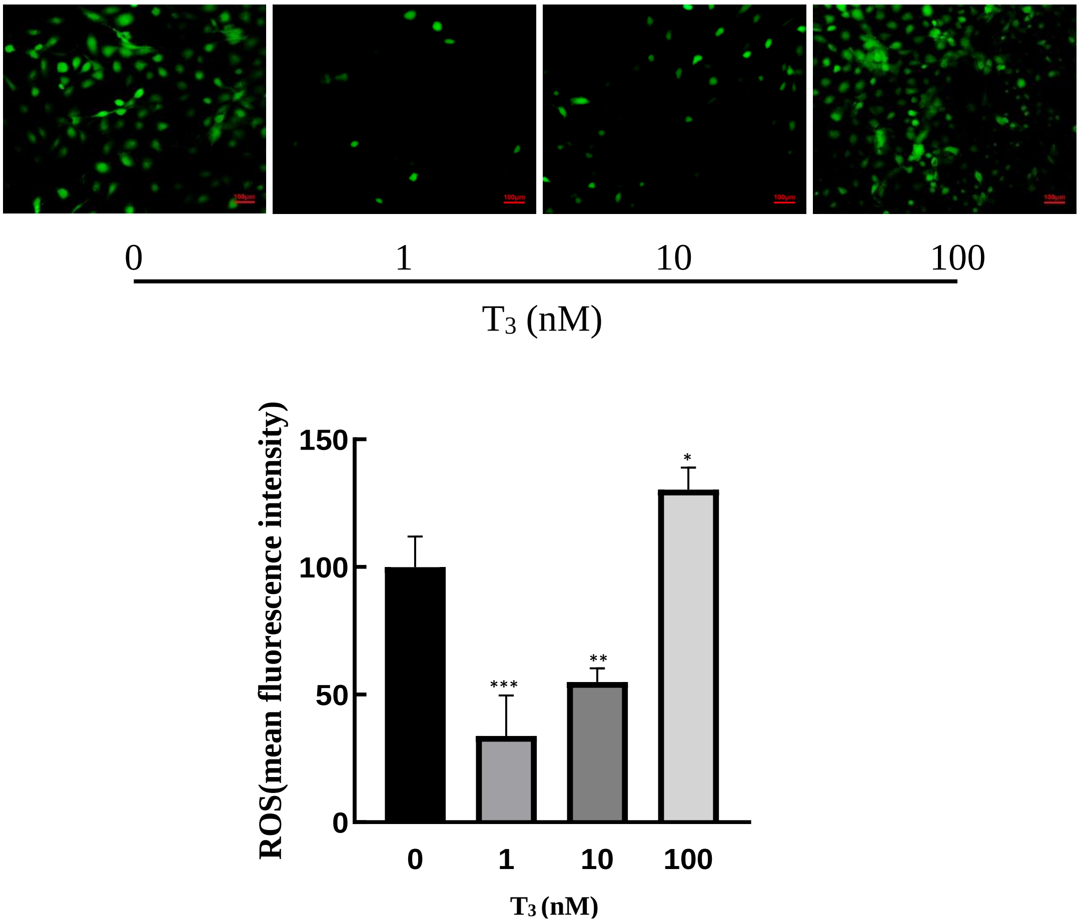

Figure S2. Effects of T_3 on ROS in rat granulosa cells. After granulosa cells were induced by H_2O_2 (100 μM) for 1 h, granulosa cells were cultured with different concentrations of T_3 (0, 1, 10 and 100 nM) for 48 h, and the ROS levels were measured. $^{***}P < 0.001$, $^{**}P < 0.01$, $^{*}P < 0.05$, compared with 0 nM group. Scale bars = 100 μm.
